# Are head and neck versus abdominal paragangliomas driven by different single nucleotide events?

**DOI:** 10.1007/s13353-025-00994-0

**Published:** 2025-07-29

**Authors:** Krzysztof Kotlarz, Katarzyna Ziemnicka, Bartłomiej Budny, Magda Mielczarek, Jakub Liu, Elżbieta Wrotkowska, Jarosław Kaznowski, Tomasz Suchocki, Jarosław Kałużny, Małgorzata Wierzbicka, Paula Dobosz, Marek Ruchała, Joanna Szyda

**Affiliations:** 1 Biostatistic Group, Department of Genetics, Wroclaw University of Environmental and Life Sciences, Wroclaw, Poland; 2 Department of Endocrinology, Metabolism and Internal Diseases, Poznan University of Medical Sciences, Poznan, Poland; 3 Charité - Institute of Biometry and Clinical Epidemiology, Berlin, Germany; 4 University Clinical Research Support Centre, Poznan University of Medical Sciences, Poznan, Poland; 5 Department of Otolaryngology and Laryngological Oncology, Poznan University of Medical Sciences, Poznan, Poland; 6 Regional Specialist Hospital Wroclaw, Research & Development Centre, Wrocław, Poland; 7 Institute of Human Genetics, Polish Academy of Sciences, Poznan, Poland; 8 Faculty of Medicine, Wroclaw University of Science and Technology, Wroclaw, Poland; 9 Department of Patomorphology and Clinical Immunology, Poznan University of Medical Sciences, Poznan, Poland

**Keywords:** Bioinformatics, Decision tree, Location heterogeneity, Paraganglioma, Single nucleotide polymorphism

## Abstract

Paragangliomas (PGLs) are a heterogeneous group of tumours of the nonepithelial neuroendocrine type, with a significant percentage being genetically determined. They can develop from cells of the parasympathetic as well as the sympathetic nervous system. Tumours located in the head and neck usually have a parasympathetic origin, whereas those in the abdomen have a sympathetic origin. The aim of this study was to determine whether the development of PGLs at both locations is associated with specific variants of genes with proven relevance for the formation of these tumours. Thirty-one patients with abdominal PGL and 16 with head and neck PGLs were analysed at 12 genes whose defects are among the most common genetic determinants of PGLs. The impact of SNPs (single nucleotide polymorphisms) on differentiation between both tumour types was assessed by fitting a decision tree and quantifying genotype effects of SNPs by the Shapley Additive Explanation metric. The study demonstrated that SNPs rs3748576 within the *KIF1B* gene and rs10060259 within the *SDHA* gene increase the probability of abdominal tumour locations, while heterozygous GA genotypes of rs2435351 located within the *RET* gene increase the probability of head and neck locations. The SNPs marked genes involved in the formation and functioning of the nervous system, but are located in introns, and thus themselves do not contribute to protein diversity. Still, intronic SNPs can indirectly affect the transcriptome by influencing alternative splicing, mRNA stability, or overlap with non-coding genes and other regulatory elements that affect transcription. Given this, it seems important to consider variants from non-coding regions in genetic analyses.

## Introduction

Paragangliomas (PGL) represent a heterogeneous group of rare tumours that belong to nonepithelial neuroendocrine neoplasms (Mete et al. 2022). PGLs exhibit a very high heritability compared to other types of tumours ranging between 0.30 and 0.35. Until now, almost 30 genes have been linked to the genetic background of PGLs (Papathomas et al. 2021). The incidence of PGLs reaches approximately 0.6 cases per 100,000 persons per year (Berends et al. 2018). Paragangliomas originate from both the parasympathetic and sympathetic nervous systems, with intra-adrenal paraganglioma called a pheochromocytoma (Mete et al. 2022). Parasympathetic PGLs are rarely hormonally active and are located primarily in the head and neck region (like carotid body, jugular, parapharyngeal, vagal or middle ear PGLs), while only 5% of tumours in this region originate from the sympathetic nervous system (Papathomas et al. 2021; Valero and Ganly 2022). Parasympathetic PGLs are usually located along the cranial and thoracic branches of the vagus and glossopharyngeal nerves (Patel et al. 2020), whereas sympathetic PGLs develop primarily in the abdominal region (Papathomas et al. 2021). So far, studies have focused on the search for new genetic variants and their association with a specific phenotype, while the aspect of possible differences in genetic predisposition to tumour development in the head and neck region and the abdominal region has not yet been studied. Whereas our study aimed to investigate whether among the genes associated with the development and course of PGLs, there are some that may also influence tumour localisation of tumours in the head and neck or in the abdominal region.

## Materials and methods

### Study population

This study considered 47 patients with no previous family history of PGL, assigned to two groups based on tumour location only in the adrenal medulla and retroperitoneal space (class 0) or with head and neck paragangliomas (class 1). All patients were diagnosed in the Department of Endocrinology, Metabolism and Internal Diseases and the Department of Otolaryngology and Laryngological Oncology at the Poznan University of Medical Sciences. For patients with abdominal PGLs, tumour hormonal activity was evaluated by measuring daily urinary excretion of metanephrines and normetanephrines, while patients with head and neck PGL were not assessed for tumour endocrine activity before surgery. Patients with pheochromocytoma and retroperitoneal paraganglioma were routinely tested during qualification for surgery. All pheochromocytomas appeared to be hormonally active, whereas retroperitoneal PGLs were hormonally inactive. The baseline characteristics of the patients studied are summarised in Table 1.

**Table 1:** Baseline characteristics of patients with PPGL

Parameter	Abdominal PPGL (0)	Head and neck PPGL (1)
Number of patients	17 female/14 male	12 female/4 male
Age at diagnosis (years)	48.6 ± 2.5 (26–74)	56.5 ±—SD 9.1 (35–70)
Pheochromocytoma	28	-
Retroperitoneal PGL	3	-
Carotid body PGL	-	4
Jugular PGL	-	1
Middle ear PGL	-	6
Vagal PGL	-	3
Parapharyngeal PGL	-	2

### Methods

#### Genetic analysis

DNA for testing was isolated from the peripheral blood of the patients. Genetic testing was conducted in the Molecular Endocrinology Laboratory in the Department of Endocrinology, Metabolism and Internal Medicine of the Poznan University of Medical Sciences. The Ion Torrent Personal Genome Machine (Ion PGM™ Thermo Fisher Scientific Inc.) was used to evaluate the custom-designed gene panel. The panel was constructed using an algorithm developed by Thermo Fisher Scientific (Ampliseq Designer, http://www.ampliseq.com), allowing for optimal design of primer pairs up to 400 bp in length. The coding regions of the following genes, described in the literature (Papathomas et al. 2021) as one of the most common defect sites associated with PGL, were selected for sequencing: *FH, KIF1B, MEN1, NF1, RET, SDHA, SDHAF2, SDHB, SDHC, SDHD, TMEM127,* and *VHL*. The final coverage of the coding sequences with amplicons varied between 95 and 100%. According to the manufacturer’s protocol, library construction and subsequent enrichment of paired DNA samples were performed using the Ion OneTouch v2 system (Thermo Fisher Scientific Inc.). The Ion PGM Sequencing 400 Kit reagents and Ion 316 v2 sequencing chips were used for direct sequencing. The average sequencing coverage varied between 110 × and 150 ×. Sequence quality filtering of the acquired reads was performed using the Torrent server and the Ion Reporter software. Further standard steps involved signal processing, read filtering, alignment to the GRCh37/hg19 human reference assembly, and genotype calling of 41 SNPs located on 6 chromosomes. Pathogenicity evaluation of coding variants was performed using the Ensembl VEP tool (McLaren et al. 2016).

#### Bioinformatic analysis

Decision trees were implemented through the ScikitLearn Python library (the DecisionTree module) to assess the impact of SNPs on patient classification (Pedregosa et al. 2011). The final tree structure was selected using the sixfold cross-validation with the optimal tree architecture based on the accuracy metric to classify the abdominal (class code 0) or head and neck (class code 1) tumour location. The impact of SNP genotypes from the final decision tree was quantified by the Shapley Additive Explanation metric (SHAP) (Lundberg et al. 2020).

## Results

**Fig. 1:**
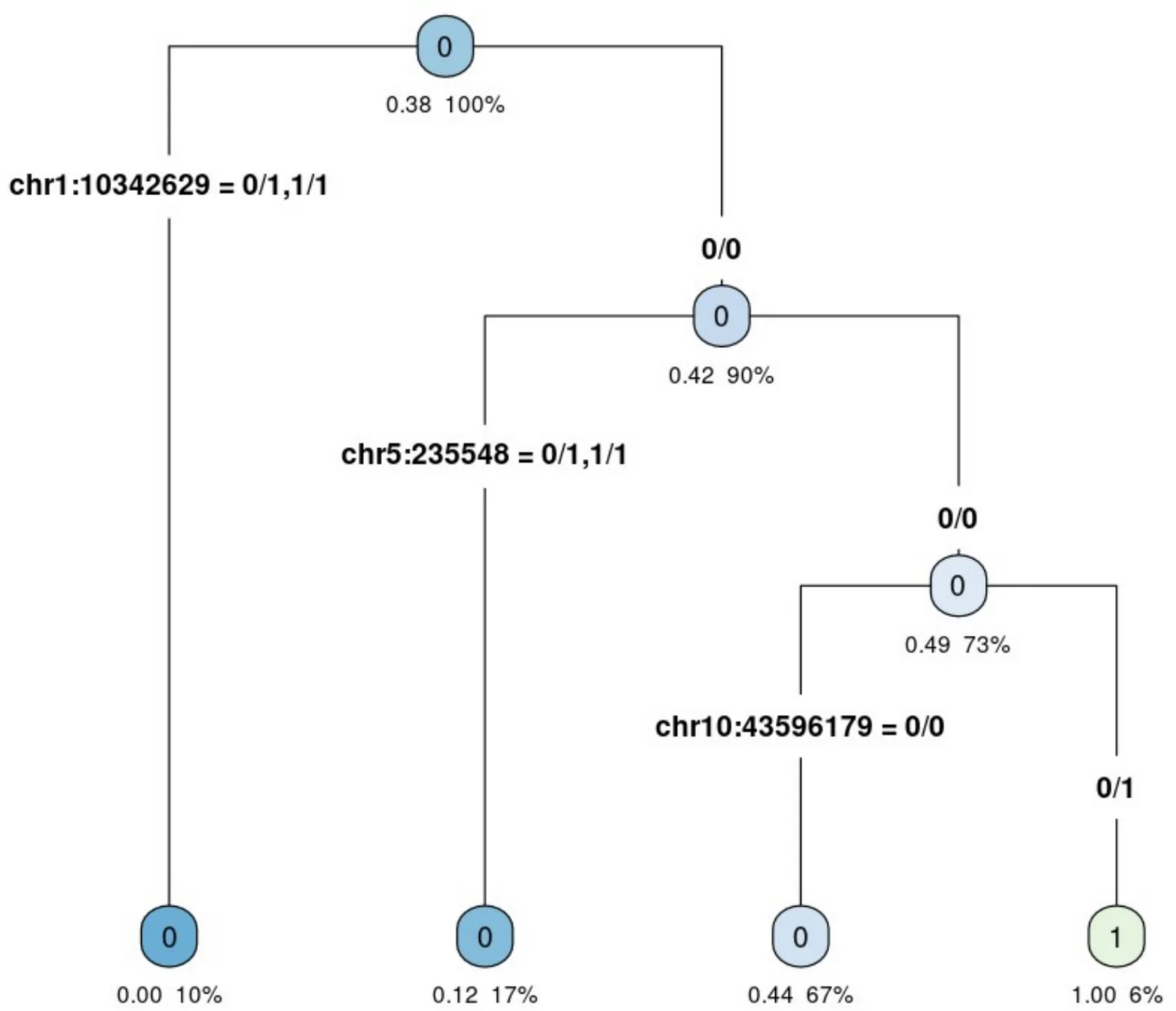
The optimal decision tree for classifying patients into the abdominal tumour location class (class 0) and the head and neck tumour location class (class 1). Next to each node, the probability of belonging to class 1 and the percentage of the remaining patients were given

**Fig. 2:**
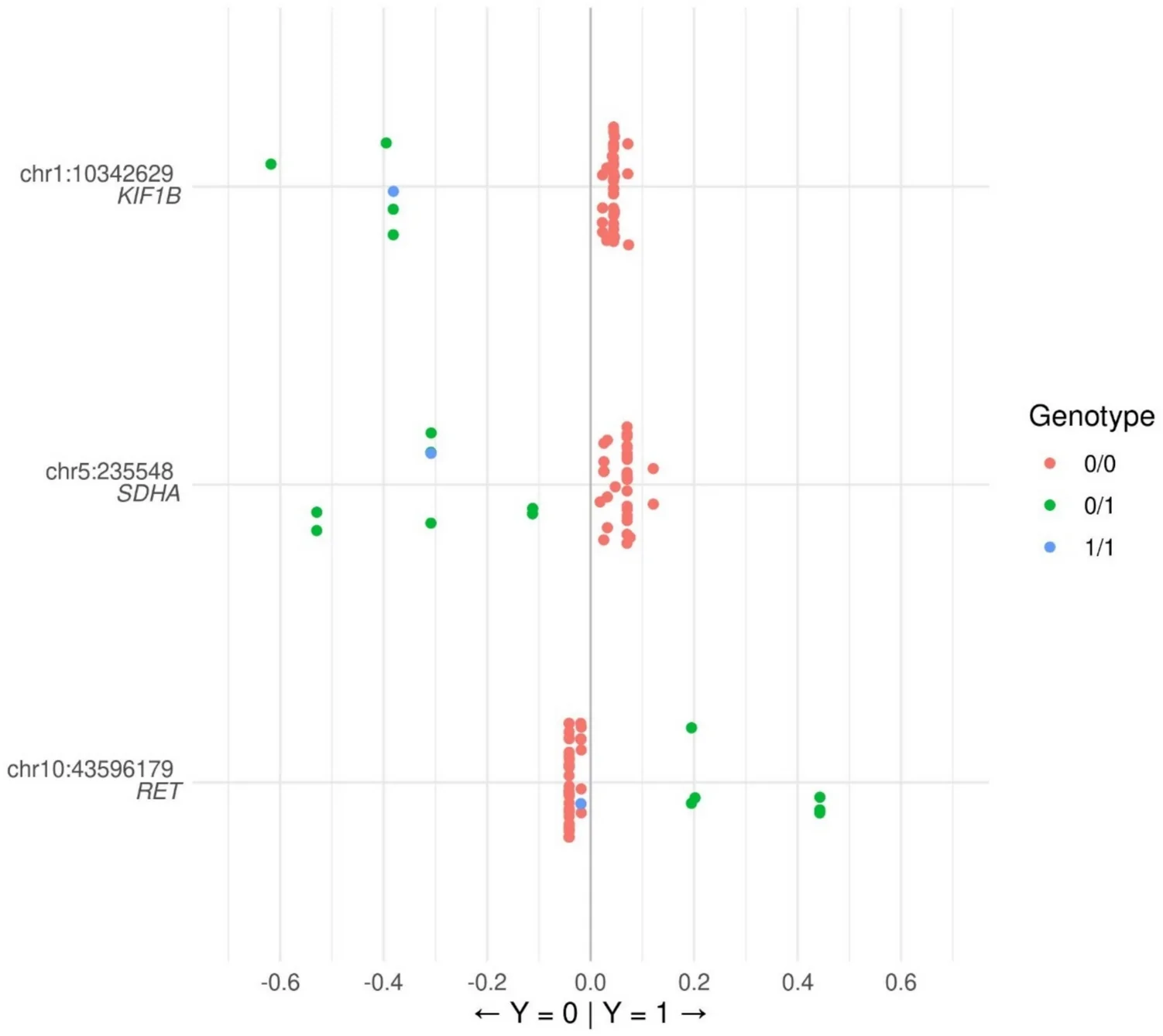
SHAP values of SNP genotypes constituting the optimal decision tree. Positive values represent an association with the head and neck tumour location class (class 1); negative values represent an association with the abdominal tumour location class (class 0)

**Fig. 3:**
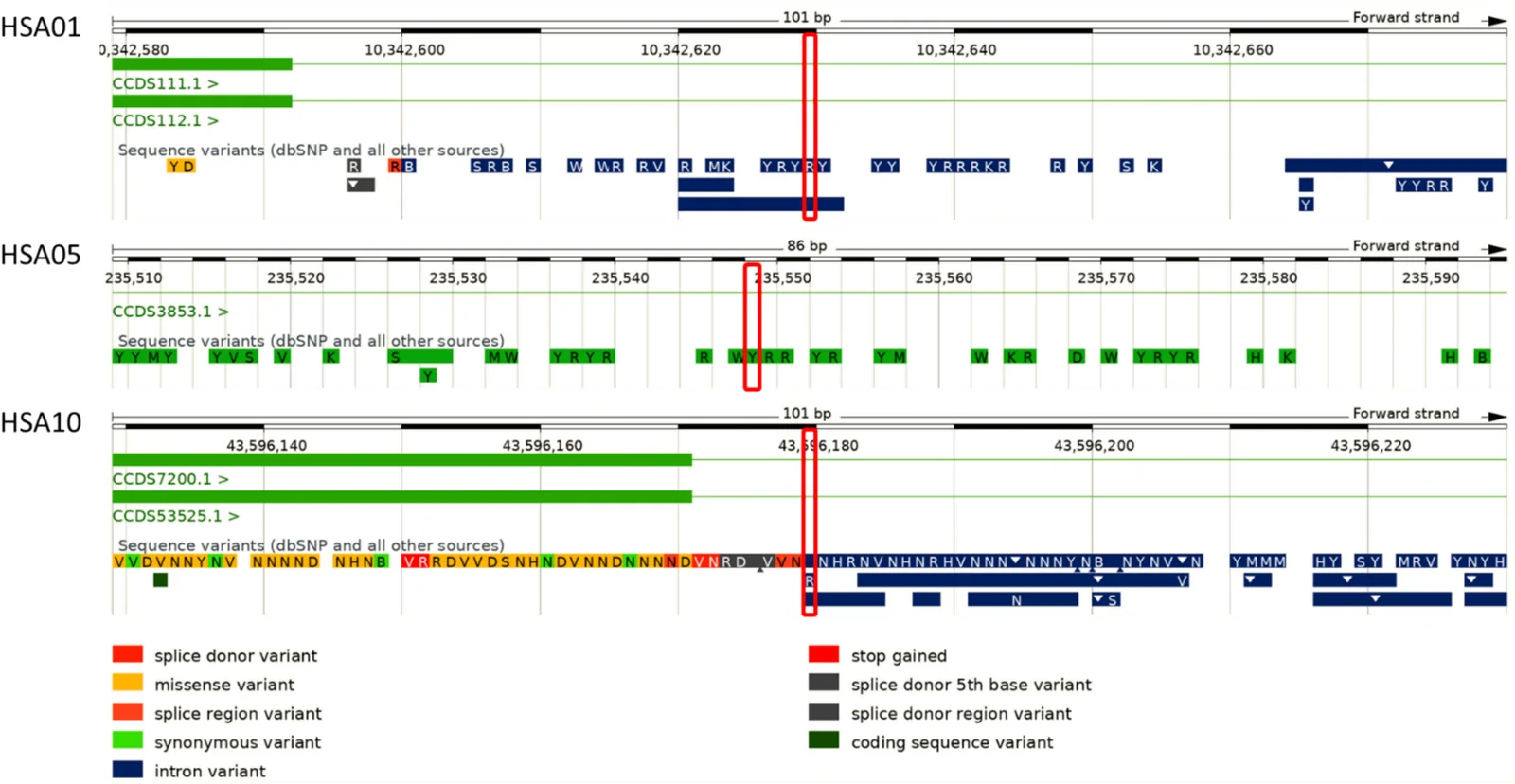
Location of chr1:10,342,629 (rs3748576), chr5:235,548 (rs10060259), and chr10:43,596,179 (rs2435351) on the GRCh37/hg19 reference assembly

For downstream analysis, only 11 SNPs with a minimum of five alternative alleles were included. The final tree architecture yielded an average classification accuracy (over the six cross-validation sets) of 72.92% (± 2.67%) for the training set and 60.42% (± 8.59%) for the validation dataset. The optimal tree architecture was visualised in Fig. 1, and the impacts of SNP genotypes on classification, expressed by SHAP values, are shown in Fig. 2. Note that positive SHAP values indicate an association with head and neck location (class 1), while negative values indicate an association with abdominal location (class 0). All three SNPs that contributed to the final decision tree were located in the non-coding part of the genes: (1) a SNP on chromosome 1 within the 15 intron of *KIF1B* (chr1:10,342,629, rs3748576), (2) a SNP on chromosome 5 within intron 9 of *SDHA* (chr5:235,548, rs10060259), and (3) a SNP on chromosome 10 within intron 2 of *RET* (chr10:43,596,179, rs2435351). The genomic location of SNPs in the GRCh37/hg19 reference assembly is visualised in Fig. 3. The functional annotation of the SNPs is summarised in Table 2.

**Table 2:** The basic characteristics of SNPs constituting the optimal decision tree

Genomic location (GRCh37)	Alleles	SNP ID	COSMIC ID	Genomic annotation	CADD score/ClinVar class
chr1:103,426	G/A	rs3748576	COSV55814192	15. intron of *KIF1B*	0.999 benign
chr5:235,548	T/C	rs10060259	-	Non-coding transcript 9. intron of *SDHA*	10.500 -
chr10:43,596,179	G/A	rs2435351	COSV60687075	2. intron of *RET*	7.850 benign

## Discussion

Paragangliomas can be caused by defects in various genes. Some of these defects are found to be common to different tumour sites (Katabathina et al. 2020; Calissendorff et al. 2022). Analysis of SNPs from the studied gene panel demonstrated that there are polymorphisms whose genotypes are indicative of tumour location. In particular, SNP rs3748576 (GA and AA genotypes) located on chromosome 1 within the *KIF1B* gene, as well as SNP rs10060259 (TC and CC genotypes) located on chromosome 5 within the *SDHA* gene, increase the probability of abdominal tumour locations, while heterozygous genotypes GA of SNP rs2435351 located on chromosome 10 within the *RET* gene increase the probability of head and neck locations. Although all three SNPs are located in introns and thus do not contribute to protein diversity, still, intronic SNPs can indirectly affect transcriptome diversity and expressivity by influencing alternative splicing (Bush et al. 2017) or stability of mRNA (Gupta et al. 2013), as well as overlap with non-coding RNA genes and other regulatory elements, especially enhancers that affect transcription (Lin et al. 2019). Moreover, the SNPs may be associated with the actual causal variants through strong linkage disequilibrium.

KIF1B (kinesin family member 1B) is a protein belonging to a family of kinesins involved in the transport of cellular materials to specified destinations (Hirokawa and Tanaka 2015). The family of these kinesins is involved in the morphogenesis, function, and survival of neuronal cells (Zhao et al. 2001). Mutations in the *KIF1B* gene in patients with PPGL have been described as germline and somatic. However, the potential importance of this gene in the development of these tumours remains elusive (Evenepoel et al. 2017). SDHA (succinate dehydrogenase complex flavoprotein A) is a subunit of succinate dehydrogenase (SDH), an enzyme that links two mitochondrial respiratory chain pathways: the Krebs cycle and oxidative phosphorylation. It is also one of the tumour suppressor genes (Gupta and Erickson 2023). Mutations in this gene have been described in patients with PGL with abdominal, cardiac, carotid body tumours, and in various other locations and origins, sympathetic or parasympathetic (Burnichon et al. 2010,Shi et al. 2023, Yoshihama et al*.* 2023, Nölting et al. 2022). Germline mutations in *SDHA* are described in approximately 10% of PPGL cases and have a low penetrance (Hanson et al. 2023). The polymorphism of the SDHA gene described in our study is located in intron 9 in a part of the gene that is close to the region coding the FAD binding domain that plays a role in maintaining the proper function of SDHA (Van Vranken et al. 2015). The *RET* (rearranged during transfection) protooncogene encodes a receptor tyrosine kinase, a membrane protein whose natural ligands are GDNF (glial-derived neurotrophic factor), neurturin, perception, and artemin. This protein controls the migration, survival, differentiation, proliferation, and maturation of vagal and sacral neural crest cells. Activation of receptor tyrosine kinase leads to the activation of numerous signalling pathways, including RAS/MAPK, PLCg, or PI3K (Bhattarai et al. 2022). RET germline mutations are detected primarily in patients with pheochromocytoma and much less frequently in those with paraganglioma (Currás-Freixes et al. 2015; Majewska et al. 2020)*.* The detected polymorphism in the RET protooncogene was located in intron 2 of the gene and therefore was within the region associated with the coding sequences of the cadherin-like domains located in the extracellular part of the receptor kinase. Genetic defects in this region may lead to impaired expression of RET on the cell surface (Takahashi et al. 2020).

The PGLs of the head and neck and the PGLs of the upper mediastinum are mainly associated with the parasympathetic system and are not hormonally active. In rare situations (5%), catecholamine-secreting head and neck PGLs that arise from either the carotid body or the carotid sympathetic chain can be identified. PGLs that develop in the lower mediastinum, abdomen, and pelvis are associated with the sympathetic nervous system and are usually hormonally active (Choi et al. 2020). In our study, we dealt with head and neck tumours, which were unknown whether they were hormonally active before surgery. However, their location indicated a high degree of probability of parasympathetic origin. Therefore, it can be assumed that these two distinguished classes, 0 and 1, represented sympathetic (abdominal PGLs) and parasympathetic (head and neck PGLs) tumours, respectively.

From a bioinformatics perspective, the major limitation of the analysis was a small study population that impedes formal significance testing of individual variants. Moreover, a small sample size contributes to a low power of causal variants detection, which typically reveal low genotype variation and may not even be polymorphic in the analysed sample. Furthermore, the group of patients with PGLs of the head and neck was almost half as numerous as those with PGL of the abdomen. Another limitation is the lack of data on the hormonal activity of tumours located in the head and neck area.

## Conclusions

By pinpointing variants located in introns, we emphasise the importance of non-coding regions in oncology, since they contain regulatory elements, as well as non-coding genome regions. Specifically, in this analysis, we demonstrated that non-protein-changing variation allows for differentiating PGL tumour location. This information may be important for predicting the risk of tumour development at a specific location in a person who is a carrier of a germline variant. However, drawing far-reaching conclusions would require increasing the study group and balancing the numbers of groups with head and neck and abdominal PGLs.

## Data Availability

The datasets generated during and/or analysed during the current study are available from the corresponding author upon reasonable request.
